# Distinguishable Plasmonic Nanoparticle and Gap Mode Properties in a Silver Nanoparticle on a Gold Film System Using Three-Dimensional FDTD Simulations

**DOI:** 10.3390/nano8080582

**Published:** 2018-07-30

**Authors:** Vasanthan Devaraj, Jong-Min Lee, Jin-Woo Oh

**Affiliations:** 1 Research Center for Energy Convergence and Technology Division, Pusan National University, Busan 46241, Korea; devarajvasanthan@gmail.com (V.D.); jongminlee1984@gmail.com (J.-M.L.); 2 Department of Nano Fusion Technology, Pusan National University, Busan 46241, Korea; 3 Department of Nanoenergy Engineering, Pusan National University, Busan 46241, Korea

**Keywords:** plasmonics, simulations, metallic nanostructures, near-field enhancement, plasmonic modes, particle on a film

## Abstract

We present a computational study of the near-field enhancement properties from a plasmonic nanomaterial based on a silver nanoparticle on a gold film. Our simulation studies show a clear distinguishability between nanoparticle mode and gap mode as a function of dielectric layer thickness. The observed nanoparticle mode is independent of dielectric layer thickness, and hence its related plasmonic properties can be investigated clearly by having a minimum of ~10-nm-thick dielectric layer on a metallic film. In case of the gap mode, the presence of minimal dielectric layer thickness is crucial (~≤4 nm), as deterioration starts rapidly thereafter. The proposed simple tunable gap-based particle on film design might open interesting studies in the field of plasmonics, extreme light confinement, sensing, and source enhancement of an emitter.

## 1. Introduction

Structures in nanoscale geometries display new properties which differ entirely from bulk behavior [1,2]. Significant examples include electron confinement in quantum dots, plasmon resonance of metallic nanoparticles, single photon emission from an isolated quantum dot, nanogenerators, and so on [3,4,5,6,7]. Of these, plasmonics is an exciting field that aims to discover a variety of properties and functions using the interaction of light and matter based on surface plasmon resonance (SPR) [8,9,10]. Upon light excitation, a collective oscillation of charge carriers arises in the interface of a metal and a dielectric with negative and positive permittivities, resulting in an SPR. These SPR-based charge carrier oscillations can be categorized into two types: localized surface plasmon resonance (LSPR) observed from confinement in a subwavelength nanostructure, and propagation of charge carriers along a planar surface referred to as surface plasmon polariton (SPP). Please note that upon exciting, both of these forms of SPR can confine the incident light at a low-subwavelength scale. Such carrier confinement leads to high enhancement of the local field and allows manipulation of light below the diffraction limit. These significant features of SPR allow plasmonic materials to generate potential applications in a variety of fields such as photonics, energy, life sciences, optics, spectroscopy, sensors, and chemistry [11,12,13,14,15,16,17,18].

Multiple characteristics of SPRs can be researched based on controlling the sizes, materials, shapes, and surface topology of the plasmonic nanomaterials, often in high precision [8,19,20,21,22]. Furthermore, by optical characterizations, it is possible to interpret the SPR of plasmonic nanomaterials based upon their geometrical parameters, surrounding dielectric medium, composition, spatial arrangements, and so on. Generally, sizeable local-field enhancements can be generated in plasmonic nanostructures based on three dynamic plasmonic designs: (i) plasmonic nanostructures which are self-tunable based on carrier control; (ii) tunable dielectric environments; and (iii) nanostructures with tunable gap sizes [23,24,25,26,27]. Of these, plasmonic nanostructures with tunable gap sizes have attracted potential interest because of dramatic electromagnetic near-field confinement and enhancement in the nanogap, which have been called hot-spots [27,28,29,30,31,32]. When plasmonic nanostructures are separated by a smaller distance in nanoscale, the nanogap effect happens in a nonradiative near-field region where SPR couples electromagnetically. This results in studying interesting plasmonic properties such as a shift in plasmonic resonances, hybrid plasmon modes, sizeable local-field enhancement (hot-spot generation), etc. Numerous studies demonstrating such large field local enhancement have been reported based on various nanostructures such as dimers, nanoparticles on a metallic film, and so on [33,34,35,36,37,38,39,40]. Of these, the nanoparticles coupled with film design has attracted significant interest recently [41,42,43,44,45,46,47,48]. Single or multiple metallic nanoparticles deposited on a metallic film exhibit resonance which is strongly localized at the gap between the nanoparticle and the film. The near-field enhancement originating from smaller sub-nanometer gap spacing in a metallic nanostructure can be significantly higher than of an individual nanoparticle structure. This property can be employed in optical sensing applications like surface-enhanced Raman spectroscopy (SERs), plasmon-enhanced photoemission, and plasmon mediated photocatalysis [8,12,23,25,31,49]. Additionally, as compared to other coupled plasmonic nanostructures, nanoparticles on a film offers several advantages such as simple fabrication, structural stability, broadband tunability, and highly reproducible optical characteristics. These superior advantages make the nanoparticle on a film design a promising platform for multiple plasmonic applications.

In the present work, we numerically investigated the plasmonic properties of a single metallic nanoparticle on a metallic mirror separated by a thin dielectric layer. From our numerical simulations, it was possible to clearly distinguish the modes/resonances originating from a nanoparticle or a gap. Such studies are essential when dealing with optical sensing and other near-field related applications.

## 2. Simulation Information

**Figure 1:**
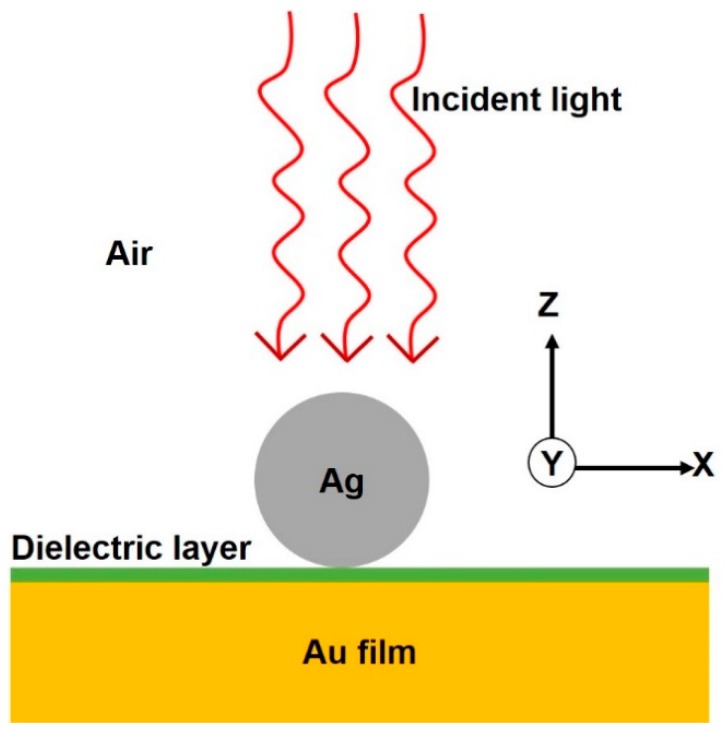
Schematic representation of a nanoparticle on a metallic mirror separated by a thin dielectric layer. Materials used in our design are as follows: gold film, a dielectric layer with index *n* = 1.5, and a silver nanoparticle.

The investigation of plasmonic nanostructures was carried out using three-dimensional finite difference time domain (3D FDTD) simulations (Lumerical solutions, Vancouver, BC, Canada). The commercial software package was installed in a server station based on an Intel Xeon CPU (40 logical processors) with 256 GB RAM capacity. The simulated particle on a film structure is shown in Figure 1. The materials employed in our design are as follows: silver nanoparticle (NP) with a diameter “*D*” of 60 nm on a gold film; Ag NP and the metallic mirror were separated by a dielectric layer (index *n* = 1.5) with a thickness of “*t*”. The plasmonic structure was surrounded by air (*n* = 1) on top. To obtain highly accurate results, mesh size of 0.2 nm was used in the simulations. The simulated region covered 300–800 nm, with a spacing of 1 nm (501 spectral points). The computational time for a single structure with 501 spectral points was ~32 h. Optical excitation from the top (“+*Z*” direction) was carried out using a broadband plane wave source. The structure was surrounded by a perfectly matched layer (PML) boundary condition in the *XYZ* directions to absorb all scattering waves. To record broadband near-field enhancement results, a box monitor was placed close to the structure. The broadband local-field enhancement spectrum was obtained from an integral volume average of *|E/E*_0_|^2^ [8,13,17]:

(1) $$Local\;field\;enhancement\;=\;\frac{∭{\left|E/E_{0}\right|}^{2}dV}{V}$$

As seen from Equation (1), a local maximum of the electric field is given by “*E*”, the incident amplitude of light source is given by “*E*_0_”, and “*V*” represent the volume. The refractive indices from the Johnson and Christy database were used for gold and silver [50]. The Lorentz-Drude dispersion model was used to model the dielectric function for the respective metallic structures [51].

(2) $$\epsilon \left(w\right)=1-\frac{f_{0}w_{p}^{2}}{w\left(w-i\Gamma_{0}\right)}+\Sigma_{j\;=\;1}^{m}\;\frac{f_{j}w_{p}^{2}}{\left(w_{j}^{2}-\;w^{2}\right)+iw\Gamma_{j}}$$

Fitting from Drude model is represented by the first term of Equation (2): plasma frequency “*w_p_*” with oscillator strength “*f*_0_” and damping constant “*Γ*_0_”. The last term in Equation (2) represents the Lorentz modification: a number of oscillations “*m*” with frequency “*w_j_*”, strength “*f_j_*”, and damping constant “*Γ_j_*”.

## 3. Results and Discussion

**Figure 2:**
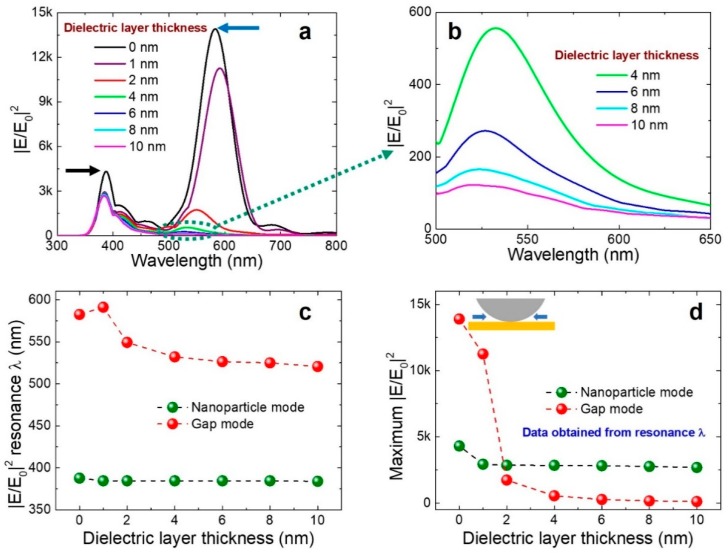
(**a**) Simulated broadband near-field enhancement spectrum as a function of dielectric layer thickness; (**b**) Magnified broadband near-field enhancement spectrum for dielectric layer thickness 4–10 nm. Resonance |*E/E*_0_|^2^ wavelengths “λ” (**c**) and respective maximum near-field enhancement (**d**) extracted from (**a**). The inset figure in (**d**) shows the presence of nanogap at both ends (blue arrows) of the metallic contact in the absence of a dielectric layer. The dashed lines are guided for eyes. Zero nanometers describe the absence of dielectric layer.

Ag NP with “*D*” = 60 nm on a thicker Au film was fixed for all simulations. To understand the NP mode and gap mode properties, we varied the dielectric layer thickness “*t*” ranging from 0 to 10 nm (0 nm represents the absence of dielectric layer). Clear separation of gap mode and NP mode can be seen in the broadband near-field enhancement spectra from Figure 2a,b as a function of dielectric layer thickness. At the longer wavelength (blue arrow mark), the resonance peak and generated |*E/E*_0_|^2^ originate from the gap plasmons, whereas, at the shorter wavelength (black arrow mark), they arise from the NP mode. The local-field enhancement (~3000 |*E/E*_0_|^2^) occurring at ~385 nm originates from silver NP and is almost constant for variable dielectric layer “*t*” (Figure 2c,d). Minimal differences in near-field enhancement properties can be seen for NP mode in the absence of the dielectric layer because of the interactions happening at metal–metal (NP-film) contact. This shows that even in the absence or presence of variable “*t*”, NP-mode-related plasmonic properties are not affected, as its origin comes from the Ag nanoparticle.

The local-field enhancement drastically reduces for the gap mode (with the blue shift in resonances) as the dielectric layer thickness increases (Figure 2c,d). At metal–metal contact (absence of dielectric layer), generation of high |*E/E*_0_|^2^ is possible because of <1 nm gaps seen at either side of metal–metal contact (Figure 2d inset). Local-field enhancement values decrease rapidly from ~14,000 to ~600 for a change in 4-nm dielectric layer thickness. This suggests that the influence of the gap mode is strong at minimal dielectric layer thickness and weakens as the dielectric layer thickness significantly enlarges. Please note that a dielectric layer thickness ~<1 nm falls under the complex quantum effect region, which will divert the discussion of our present work [6,28]. This study shows critical importance in analyzing NP mode and gap mode for a particle on a film design. Plasmonic properties related to NP mode are not seriously affected, either in the presence or absence of a dielectric layer, as its origin comes from the nanoparticle itself. To clearly analyze NP mode optical properties, a reasonably large dielectric layer thickness of ~≥10 nm) is enough. However, for the gap mode, the small dielectric layer thickness is critical, as local-field enhancement deteriorates for increasing dielectric layer “*t*”.

**Figure 3:**
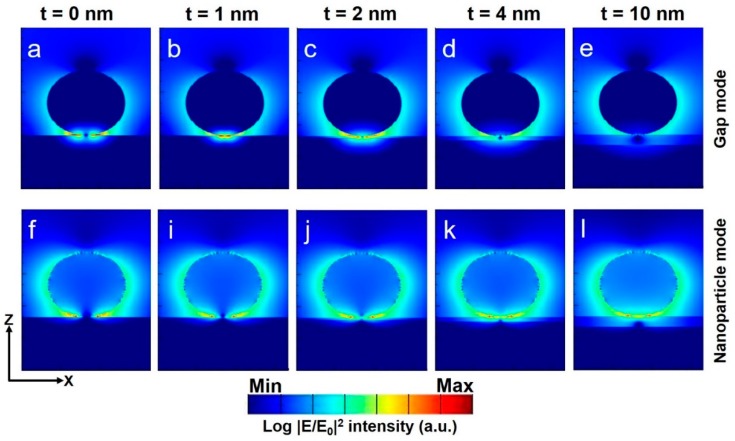
Cross-sectional (*XZ*) electric field amplitude profiles obtained from the peak resonances of gap modes (**a**–**e**) and nanoparticle modes (**f**–**l**) as a function of dielectric layer thickness “*t*”. The color ranges are in log scale.

Figure 3 shows the cross-sectional *XZ* electric field amplitude profiles which support our above-discussed optical interpretation. The electric field amplitude profiles for gap modes (Figure 3a–e) obtained at their respective resonance wavelengths (Figure 2c) as a function of dielectric layer thicknesses of 0, 1, 2, 4, and 10 nm, respectively. Similarly, Figure 3f–l) shows the electric field amplitude profiles taken at the resonance wavelength for nanoparticle mode of the structure for similar dielectric layer thickness. The electric field intensity for a gap mode for “*t*” = 0 nm or 1 nm is highly enhanced at the NP-film or NP-dielectric layer–film interface. As “*t*” increases further, clear deterioration of near-field intensity can be seen at the interfaces. However, for the NP modes, no such behavior is observed, and electric field intensity looks similar with all simulated dielectric layer “*t*”. To study the properties of NP mode only, a reasonably large enough dielectric layer thickness (~≤10 nm) will be enough. Critical near-field intensity changes based on gap modes by the following conditions: at smaller “*t*” ~ ≤4 nm, plasmonic properties arising from the gap mode are dominant and deteriorate when the “*t*” increases further. The significant difference from Au NP on an Au film (Au–Au) system compared with our Ag NP on an Au film is the clear distinguishability of gap and NP modes. In the case of an Au–Au system, there is no clear separation between NP and gap modes, and the possibilities of distinguishing both modes would be minimal (see supporting information Figure S1). For a cross-check, we simulated an Au NP on Ag film system and analyzed the local-field enhancement properties as function of a dielectric layer thickness for a comparison. No clear separation between NP and gap modes were observed, as seen from the Au–Au system (see supporting information Figure S2 for details). Thus, our Ag NP on an Au film design shows significant optical properties in analyzing individual and distinguishable mode properties (gap and NP) clearly, which can possibly be used in interesting applications.

Please note that fabrication of previously reported tunable gap structures to clearly distinguish the gap and nanoparticle modes (such as dimers with geometries involving spheres, disks, cubes, nanorods, bow-tie antennae, and so on) on a film requires few complex processes to start. Smaller interparticle (~<5 or 10 nm) distance between such dimers on a film is difficult to achieve in single or a few fabrication attempts. Our single nanoparticle on a film with a thin dielectric layer in between (which is electromagnetically similar) is a simple design to fabricate with sub-nanometer gaps below <5 nm [41]. It is possible to control the dielectric layer thickness precisely and, thus, observing gap mode properties at minimal dielectric layer “*t*” (~1 or 2 nm) is very much possible from e-beam deposition methods. One of the important observations from our work is the clear distinguishability between nanoparticle and gap modes. Such analyses of individual and clearly distinguishable mode properties (nanoparticle and gap) are significant as they will find interesting applications in the field of selective or tunable photonics or plasmonic-related devices. With such advantages, the particle on a film system helps us to investigate specific optical properties at a precise scale.

## 4. Conclusions

In summary, we reported a simple plasmonic nanostructure design (particle on a film) to investigate in-depth plasmonic properties such as gap and nanoparticle modes. Our three-dimensional finite difference time domain simulation studies showed the influence of dielectric layer thickness when coming towards the significant distinguishability between nanoparticle and gap modes. Strong gap mode influences were observed at minimal dielectric layer “*t*” (≤4 nm) and started deteriorating with further increases in “*t*” based on our design. In the presence of a large enough dielectric layer thickness, it is possible to analyze the nanoparticle mode independently and provide essential insights to analyze the individual optical behavior. The proposed plasmonic design is easy to fabricate even with a thinner dielectric layer on a metallic film, enabling detailed gap-mode-based studies. The proposed design approach opens the path for a variety of applications in the field of plasmonics, optics, sensors, and photonics.

## Supplementary Materials

The following are available online at http://www.mdpi.com/2079-4991/8/8/582/s1, Figure S1: 3D FDTD simulation results of Au NP on Au film in presence of dielectric layer. Figure S2: 3D FDTD simulation results of Au NP on Ag film in presence of dielectric layer.
