# Supplementary material for: Distinguishable Plasmonic Nanoparticle and Gap Mode Properties in a Silver Nanoparticle on a Gold Film System Using Three-Dimensional FDTD Simulations
Source: Nanomaterials (Basel). 2018 Jul 30;8(8):582. doi: 10.3390/nano8080582 (PMC6116242; doi:10.3390/nano8080582)
Supplement: Supplementary file 1 [file nanomaterials-08-00582-s001.pdf]

# Supporting Information: Distinguishable Plasmonic Nanoparticle and Gap Mode Properties in a Silver Nanoparticle on a Gold Film System Using Three-dimensional FDTD Simulations

**Vasanthan Devaraj [1], Jong-Min Lee [1] and Jin-Woo Oh [1,2,3,*]**

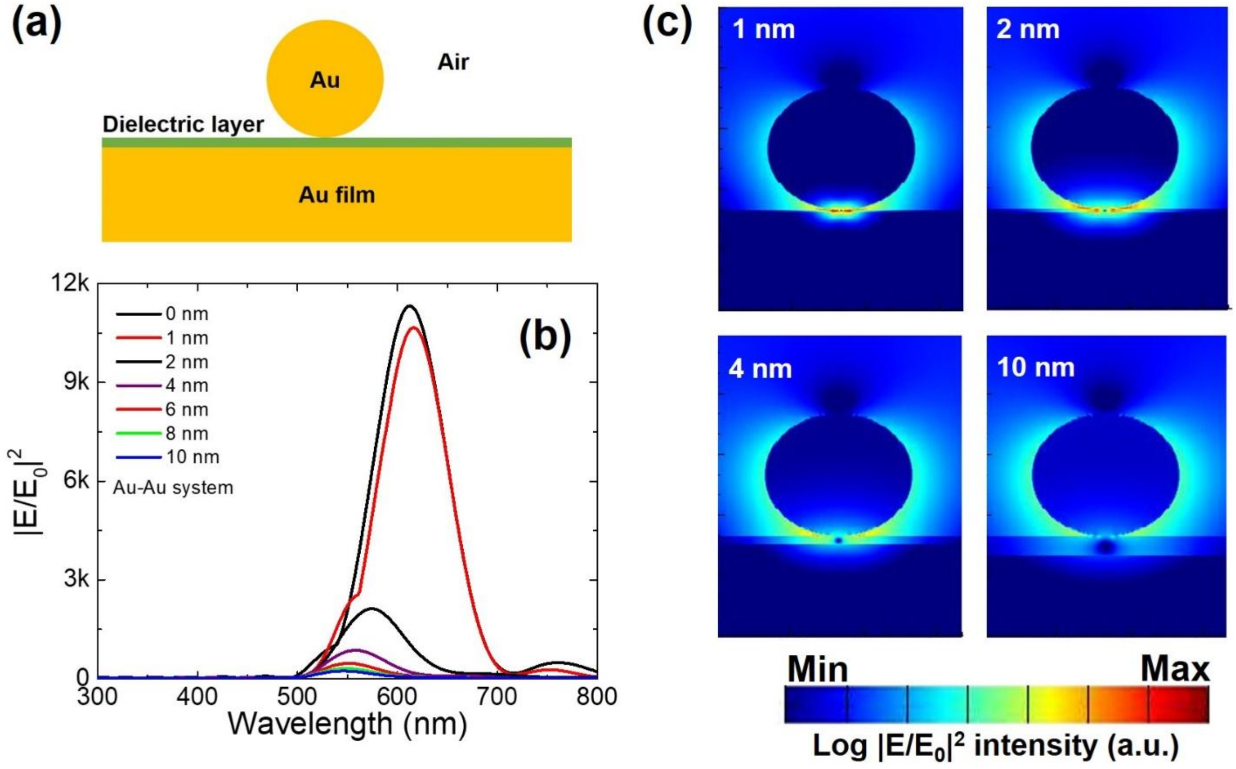


**Figure S1.** (**a**) Au NP on Au film in presence of dielectric layer. (**b**) Broadband local field enhancement profiles for dielectric layer thickness 0 – 10 nm. (**c**) Electric field amplitude profiles for dielectric layer thickness mentioned in respective profile images taken at peak resonant $|E/E_0|^2$ wavelength. At smaller dielectric layer thickness (~ 1–4 nm), gap and nanoparticle modes are not clearly distinguishable between each other as seen from respective electric field profiles (Figure S1c). As the thickness of dielectric layer increases further, gap mode weakens or becomes negligible, and nanoparticle mode is primarily seen (for example electric field amplitude profile for 10 nm thickness).

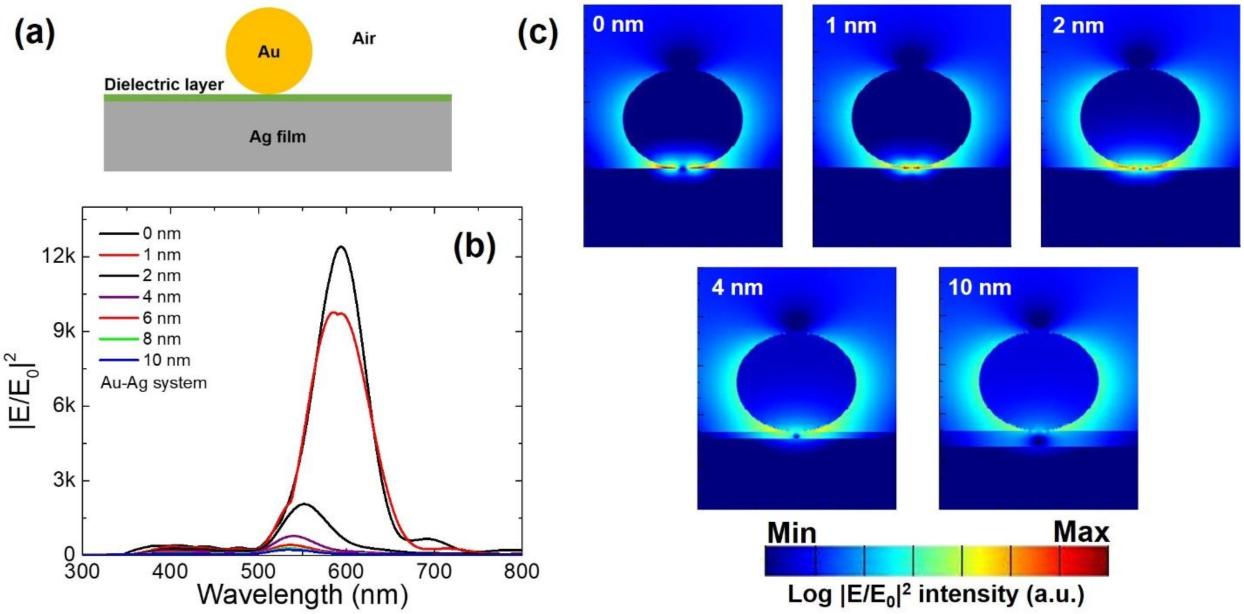


**Figure S2.** (**a**) Au NP on Ag film in presence of dielectric layer. (**b**) Broadband local field enhancement profiles as a function of dielectric layer thickness (0–10 nm). (**c**) Selective electric field amplitude profiles for dielectric layer thickness mentioned in inset taken at peak resonant $|E/E_0|^2$ wavelength. A similar non-distinguishable modes as observed in Au-Au system can be seen from obtained respective electric field amplitude profiles.
